# Determinants of abortion views among reproductive age women in Georgia 2023–2024

**DOI:** 10.1371/journal.pone.0335370

**Published:** 2025-11-12

**Authors:** Madeline Chandler, Jasmin A. Darville, Stephanie M. Eick

**Affiliations:** 1 Gangarosa Department of Environmental Health, Rollins School of Public Health, Emory University, Atlanta, GeorgiaUnited States of America; 2 Department of Epidemiology, Rollins School of Public Health, Emory University, Atlanta, GeorgiaUnited States of America; 3 School of Medicine, Emory University, Atlanta, GeorgiaUnited States of America; Georgetown University Medical Center, UNITED STATES OF AMERICA

## Abstract

**Background:**

Abortion is a continually debated legislative issue in the United States. We aimed to assess opinions toward abortion access amongst reproductive age adult women in Georgia, a state where abortion is banned after the detection of embryonic cardiac activity (around 6 weeks).

**Methods:**

Participants were enrolled in the cross-sectional Policies and Social Stress (PASS) Study (N = 177). Sociodemographic characteristics, political orientation, religious characteristics, county of residence, and abortion views were assessed using self-reported questionnaires. Regarding abortion views, participants were asked about their opinion on the legality of abortion generally, and at 6, 14, and 24 weeks. We used logistic regression to examine whether sociodemographic factors, political orientation, religious characteristics, and county of residence were associated with odds of thinking abortion should be legal or illegal for each weeks’ gestation.

**Results:**

Most participants (84%) reported supporting the legality of abortion in all or most cases. Though, that support decreased for specific weeks’ gestation (6 Weeks: 76%; 14 Weeks: 60%; 24 Weeks: 31%). Conservatives and moderates had higher odds of thinking abortion should be generally illegal compared to liberals (odds ratio [OR]=10.2, 95% confidence interval [CI]=4.1–27.3). Those who attended religious services more often and those who resided outside of the Atlanta area were more likely to believe abortion should be illegal compared to reference groups (OR=7.2, 95% CI = 3.0–17.9; OR=6.0, 95% CI = 2.5–16.3, respectively). However, the differences between groups attenuated as the weeks of pregnancy increased.

**Conclusions:**

In a sample of reproductive age women in Georgia, we observed that opinions regarding the legality of abortion were nuanced with regards to gestational age. Fewer participants supported abortion access after fetal viability (i.e., around 24 weeks). Further, attitudes differed mostly along political, religious, and geographic lines.

**Implications:**

While 76% of adult women in our sample supported abortion access at six weeks of pregnancy, less than one-third supported abortion at 24 weeks of pregnancy. These findings suggest that Georgia’s state policy that limits abortion after six weeks’ gestation does not reflect the views of the women in our sample.

## Introduction

Abortion, defined as a medical intervention that terminates a pregnancy, is one of the most consistently debated legislative issues in the United States (U.S.). Women are uniquely impacted by access to abortion care. Policies surrounding the specifications and limitations of abortion care vary by state, however the right to an abortion was protected nationally until the 2022 Supreme Court decision, *Dobbs v. Jackson* (i.e., Dobbs decision) which overturned *Roe v. Wade*.

Given the importance of abortion in the national conversation, it is critical to understand what the main drivers of these views are. Research aimed at understanding drivers of public opinion in the U.S. find that while public support of abortion has hovered around 60% since 1995 (and stood at 63% in 2024), views vary by political affiliation, religious identity, and other demographic characteristics [1–5]. For example, a 2024 survey of U.S. adults conducted by the Pew Research Center found that 85% of liberal-leaning persons support abortion in all or most cases and only 41% of conservative-leaning individuals saying the same [2].

Though much research has examined the relationship between demographic characteristics and abortion, there is less of an understanding of how support for abortion differs across gestational age. For example, a survey of a nationally-representative sample found that even those who identify as “pro-life” or “pro-choice” have inconsistent beliefs when questioned about specific scenarios related to the health of the mother, health of the fetus, and circumstances of the pregnancy [4]. Prior research on abortion at varying gestational ages demonstrates an attrition in abortion support as gestational age increases, suggesting that when polled broadly about abortion opinion, people are likely to think about abortion in early pregnancy [6]. Also, research has shown that survey design can impact responses relating to abortion depending on wording and placement of survey items [7].

The present analysis is the first study since the Dobbs decision to investigate abortion views within Georgia among reproductive aged adult women, which allows for a more robust examination of perspectives on abortion policy within a highly politically polarized geographic area. Given the lack of any federal policy on abortion, policies are now most consequential at the state level. Currently, Georgia prohibits any abortion after the detection of embryonic cardiac activity, which typically occurs around six weeks of pregnancy (Georgia House Bill 481). Somewhat uniquely, HB481 also includes a personhood clause, which defines a fetus as a person with legal rights. It is important to gain an understanding of abortion views among reproductive age adult women in Georgia since they are likely to be the most directly impacted by these shifts in policies. The aim of this study was to capture these abortion views and their determinants during a time of heightened political discourse, the year leading up to the 2024 US Presidential Election.

## Methods

### Study population

Participants in this analysis were enrolled in the Policies and Social Stress (PASS) Study, a cross-sectional study designed to identify sources of stress among adult women of reproductive age (n = 177). This study was approved by the Emory University Institutional Review Board, approval number STUDY00006059. Inclusion was as follows: self-identify as a woman, between 18–40 years of age, able to communicate in English, no history of hysterectomy, and residing in Georgia. Information on gender identity was not collected as part of our study and both biological females and trans women were eligible to participate. However, we use the term ‘women’ throughout for simplicity. Participants were recruited using targeted social media ads on Facebook and Instagram between November 10, 2023 and November 25, 2024. Targeted ads were based on age (18–40 years of age) and location (Georgia) and included a link to the screening form. Individuals who were deemed eligible to participate in our study after completion of the screening form were sent a personalized link to a REDCap survey, where they were able to complete self-reported questionnaires. Additional information regarding recruitment and a description of the study population is provided elsewhere [8]. Participants completed electrotonic written informed consent prior to receiving the link to the questionnaires.

### Independent variables

#### Demographic characteristics.

Participants were asked a variety of questions regarding their sociodemographic information, including their age (in years), race (White, Black/African American, Asian, Other), ethnicity (Non-Hispanic or Latina, Hispanic or Latina), marital status (married or living with partner, single), education (≥college degree, < college degree), annual household income (<$55,000, ≥ $55,000), employment (employed part or full time, unemployed), current student (yes, no), ever been pregnant (yes, no). Due to sparse data, racial categories were collapsed into White, Black, and Other for analysis.

#### Political orientation.

Participants self-reported their political orientation, with possible answer options including “Liberal,” “Lean liberal,” “Moderate,” “Lean conservative,” or “Conservative.” To maximize statistical power across categories, “Moderate” (n = 21), “Lean conservative” (n = 8), or “Conservative” (n = 15) were collapsed into one category, with those responding “Lean liberal” (n = 38) or “Liberal” (n = 85) in another.

#### Religious characteristics.

Religious identity and religious service attendance were assessed using the questions: “Do you consider yourself a religious person?” (yes, no), and “Aside from weddings and funerals, how often do you attend religious services?” (more than once a week or once a week versus once or twice a month, a few times a year, seldom, or never).

#### County of residence.

Participants were asked to indicate their county of residence. We categorized participants as being residents of the Atlanta metro area if their county of residence was among the 11 counties that the Atlanta Regional Commission considers to be within the Atlanta metro [9]. Otherwise, participants were considered to live outside of the Atlanta metro area.

### Dependent variables

#### Abortion views.

Our primary outcome of interest was abortion views, which was assessed using the following question: “Do you think abortion should be…” Answer options included: “legal in all cases”, “legal in most cases”, “illegal in most cases”, and “illegal in all cases”. For analyses, responses were combined into two categories (legal in all/most cases and illegal in all/most cases) due to sparse data across categories.

Other questions to further clarify participants’ views were also asked with scenario-based prompting. Specifically, participants were asked about their view on abortion at 6 weeks, 14 weeks, and 24 weeks using the following questions. These questions were adapted from the Pew Research Center [10].

“Approximately 6 weeks into a pregnancy is about when cardiac activity (sometimes called a fetal heartbeat) can be detected, and before many women know they are pregnant. Do you think abortion at that point should be…”

“As you may know, a typical pregnancy lasts for roughly 40 weeks. Thinking specifically about a pregnancy that is 14 weeks along (roughly at the end of the first trimester), do you think abortion at that point should be…”

“Approximately 24 weeks into a pregnancy (near the end of the second trimester) is about when a healthy fetus could survive outside the woman’s body with medical attention. Do you think abortion at that point should be…”

Answer options for all questions included “legal in this case,” “illegal in this case,” or “it depends.” For this analysis, “it depends” and “illegal in this case” were combined into one category due to the relatively few numbers of participants reporting “it depends.”

### Statistical analysis

We began by examining the distributions of independent variables and dependent variables in our study population by calculating descriptive statistics. To evaluate whether demographic characteristics, political orientation, and religious characteristics are associated with increased odds of thinking abortion should be illegal (overall, week-specific scenarios), we conducted a series of logistic regression models. The reference group for all models was “legal in most or all cases.” In our primary analysis, we present unadjusted odds ratio due to collinearity concerns between certain variables (e.g., educational attainment and student status). In secondary analyses, we re-ran all models minimally adjusting for age, education status, marital status, and prior pregnancy.

## Results

There were 177 participants included in our analytic sample (Table 1). Of these participants the mean age was 29 years (standard deviation [SD]=6) and most participants self-identified as White (45%) or Black/African American (37%). Our study population was overwhelmingly non-Hispanic or Latina (92%). The majority of participants identified as liberal (74%). Less than half of participants considered themselves to be a “religious person” (46%) and only 27% attended religious services weekly or more often. Participants in this study primarily resided in the Atlanta metro area of Georgia (61%). Notably, with regards to abortion views, while 84% say that generally abortion should be legal in all or most cases, the number of participants saying that abortion should be legal decreases across the presented scenarios, with 76% saying abortion should be legal at 6 weeks, 60% at 14 weeks, and 31% at 24 weeks (Table 1).

**Table 1:** Distribution of sociodemographic characteristics and scenario-specific abortion views within the Policies and Social Stress (PASS) Study (N = 177).

Characteristic	N (%), Mean (SD), or [Min, Max]
Age	29 (6)
Range	[18, 40]
Missing	1
Race	
White	80 (45%)
Black	65 (37%)
Asian	21 (12%)
Other	10 (5.7%)
Missing	1
Ethnicity	
Not Hispanic or Latina	161 (92%)
Hispanic or Latina	14 (8.0%)
Missing	2
Political Orientation	
Liberal	123 (74%)
Moderate or Conservative	44 (26%)
Missing	10
Educational Attainment	
< College degree	54 (31%)
≥ College degree	122 (69%)
Missing	1
Household Income	
< $54,999	65 (39%)
≥ $55,000	103 (61%)
Missing	9
Employment Status	
Unemployed	46 (26%)
Employed part or full time	129 (74%)
Missing	2
Student	
Not a student	121 (69%)
Current student	55 (31%)
Missing	1
Marital Status	
Married or living with partner	88 (51%)
Single	85 (49%)
Missing	4
Ever Been Pregnant	
Been pregnant before	82 (47%)
Never been pregnant	93 (53%)
Missing	2
Religious Identity	
Non-religious person	92 (54%)
Religious Person	78 (46%)
Missing	7
Religious Service Attendance	
Monthly or less often	129 (73%)
Once a week or more often	47 (27%)
Missing	1
County	
Metro Atlanta County	107 (61%)
Not in Metro Atlanta	69 (39%)
Missing	1
Abortion View – Overall	
Legal in most/all cases	147 (84%)
Illegal in most/all cases	27 (16%)
Missing	3
Abortion View – 6 Weeks	
Legal in this case	132 (76%)
Illegal or it depends	41 (24%)
Missing	4
Abortion View – 14 Weeks	
Legal in this case	103 (60%)
Illegal or it depends	70 (40%)
Missing	4
Abortion View – 24 Weeks	
Legal in this case	53 (31%)
Illegal or it depends	117 (69%)
Missing	7

When examining whether certain demographic characteristics were associated with differences in abortion views, we observed that almost all associations were not statistically significant, though a few patterns are notable (Table 2). Generally, those who have never been pregnant (vs. have been pregnant) and current students (vs. non-students) are much less likely to believe abortion should be illegal in any situation. Furthermore, the logistic regression models for demographic characteristics demonstrate that abortion views are not consistent across the presented scenarios. Corresponding unadjusted odds ratios are shown in Table 2 and adjusted odds ratios are shown in S1 Table.

**Table 2:** Associations between sociodemographic characteristics and odds of self-reporting that abortion should be illegal according to different scenarios, estimated using unadjusted logistic regression models in the Policies and Social Stress (PASS) Study.

	Overall		6 Weeks		14 Weeks		24 Weeks	
	OR	95% CI	OR	95% CI	OR	95% CI	OR	95% CI
Age	1.02	(0.96, 1.09)	1.04	(0.99, 1.11)	1.03	(0.98, 1.09)	1.05	(0.99, 1.11)
Race								
White	—	—	—	—	—	—	—	—
Black	1.60	(0.68, 3.80)	1.43	(0.66, 3.12)	4.44	(2.20, 9.25)	2.11	(1.00, 4.66)
Other	0.19	(0.01, 1.01)	1.08	(0.38, 2.85)	2.29	(0.94, 5.57)	0.92	(0.38, 2.26)
Ethnicity								
Not Hispanic or Latina	—	—	—	—	—	—	—	—
Hispanic or Latina	0.89	(0.13, 3.52)	0.85	(0.19, 2.91)	0.55	(0.15, 1.73)	1.01	(0.31, 3.87)
Marital Status								
Married or living with partner	—	—	—	—	—	—	—	—
Single	0.47	(0.19, 1.08)	0.51	(0.24, 1.04)	0.47	(0.25, 0.88)	0.71	(0.36, 1.37)
Household Income								
< $54,999	—	—	—	—	—	—	—	—
≥ $55,000	1.33	(0.57, 3.29)	1.58	(0.75, 3.49)	1.56	(0.82, 3.01)	1.85	(0.93, 3.66)
Ever Been Pregnant								
Been pregnant before	—	—	—	—	—	—	—	—
Never been pregnant	0.20	(0.07, 0.50)	0.26	(0.12, 0.54)	0.17	(0.08, 0.33)	0.20	(0.09, 0.41)
Employment Status								
Unemployed	—	—	—	—	—	—	—	—
Employed part or full time	0.81	(0.34, 2.11)	0.79	(0.37, 1.77)	0.88	(0.44, 1.78)	0.60	(0.26, 1.29)
Student								
Not a student	—	—	—	—	—	—	—	—
Current student	0.33	(0.09, 0.93)	0.64	(0.28, 1.38)	0.40	(0.19, 0.81)	0.42	(0.21, 0.83)
Educational Attainment								
< College degree	—	—	—	—	—	—	—	—
≥ College degree	0.71	(0.30, 1.72)	0.79	(0.38, 1.70)	0.97	(0.50, 1.91)	0.72	(0.34, 1.48)

— Indicates reference group.

OR, odds ratio; CI, confidence interval.

Note: Models are estimating the odds of believing that abortion should be illegal or that “it depends” in this case versus the belief that abortion should be legal in this case.

**Fig 1:**
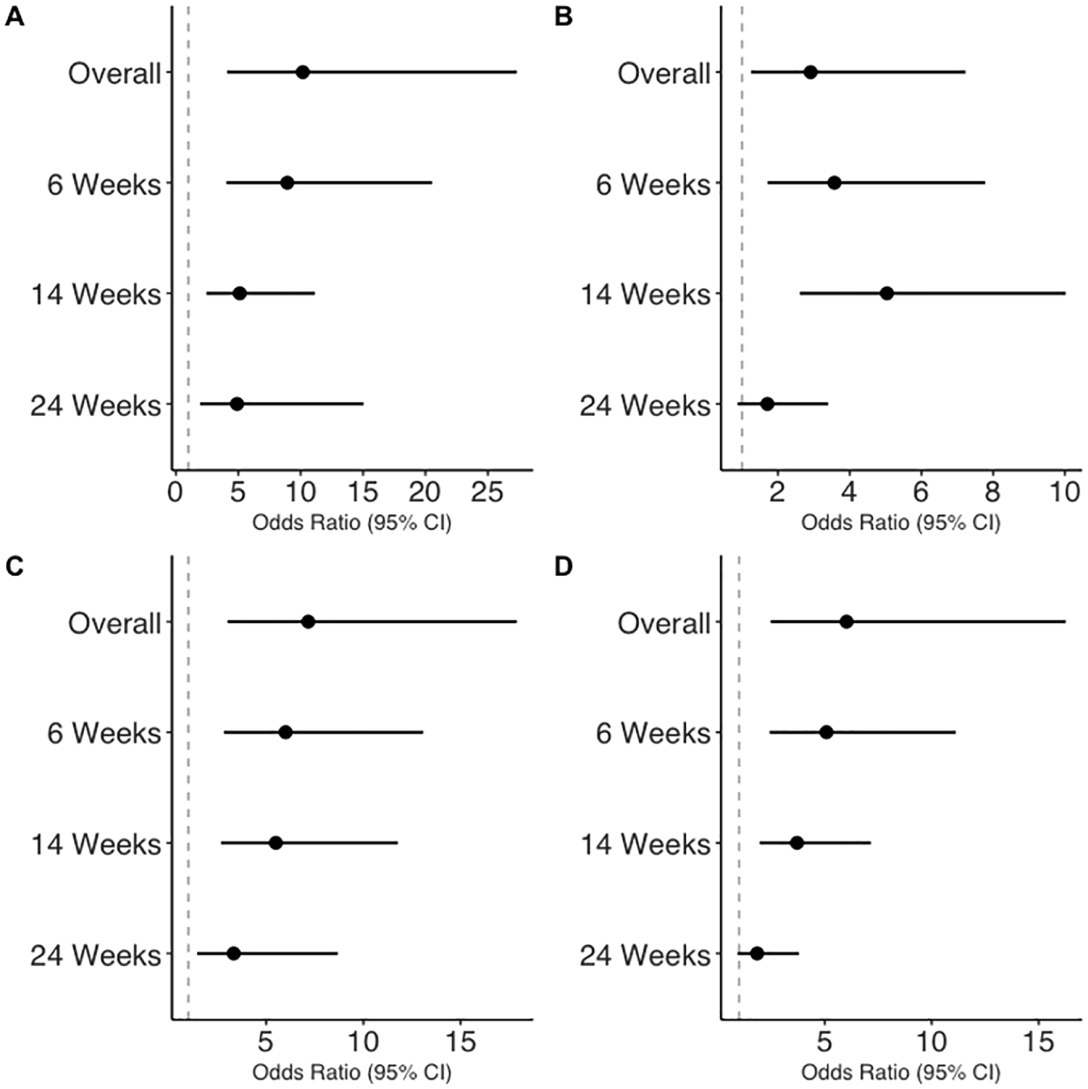
Associations between political, religious, and geographic characteristics and abortion views for week-based scenarios, estimated using unadjusted linear regression models. Exposures are as follows: (A) political orientation (moderate or conservative, *ref: liberal***), (B) religious identity (religious person,** *ref: non-religious person***), (C) religious service attendance (once a week or more,** *ref: once per month or less***), and (D) county of residence (non-Atlanta metro county,** *ref: Atlanta metro county***).** The models are estimating the odds of believing that abortion should be illegal or that “it depends” in this case (vs. abortion should be legal in this case).

We observed that political orientation, religious identity, religious service attendance, and county of residence were all strongly associated with abortion views in unadjusted models (Fig 1 and S2 Table). Notably, these associations were variable across the week-based scenarios, which mirrors our findings from models which included sociodemographic characteristics as the independent variables. For example, conservatives and moderates had much higher odds of thinking abortion should be illegal compared to liberals (OR_overall_ = 10.2, 95% CI = 4.1, 27.3). Notably, the association weakened in magnitude across the specific scenarios (OR_6-weeks_ = 8.9, 95% CI = 4.0, 20.5; OR_14-weeks=_5.1, 95% CI = 2.5, 11.1; OR_24-weeks_ = 4.9, 95% CI = 1.9, 15.0). Those who attended religious services once a week or more were also more likely to believe that abortion should be illegal compared to those who attended less often (OR_overall_ = 7.2, 95% CI = 3.0, 17.9), however that association also attenuated across increasing gestational weeks (e.g., OR_6-weeks_ = 6.0, 95% CI = 2.8, 13.1 versus OR_24-weeks_ = 3.3, 95% CI = 1.5, 8.7). While these same patterns persisted in minimally adjusted models, some associations were attenuated to non-significance (S1 Table).

This pattern was also observed in models which considered county of residence as the independent variable. Specifically, those who lived outside of the Atlanta metro area were more likely to believe that abortion should be illegal (OR_overall_ = 6.0, 95% CI = 2.5, 16.3), but the strength of that association decreases with the week-based scenarios (OR_6-weeks_ = 5.1, 95% CI = 2.4, 11.1; OR_14-weeks_ = 3.7, 95% CI = 2.0, 7.2; OR_24-weeks_ = 1.8, 95% CI = 0.9, 3.8). While overall, those who identify as religious are more likely to believe that abortion should be illegal compared to non-religious people (OR_overall_ = 2.9, 95% CI = 1.3, 7.2), the more specific scenarios follow a less consistent pattern (OR_6-weeks_ = 3.6, 95% CI = 1.7, 7.8; OR_14-weeks_ = 5.0, 95% CI = 2.6, 10.0; OR_24-weeks_ = 1.7, 95% CI = 0.9, 3.4). Sample sizes for these models are provided in S3 Table.

## Discussion

In this study, we aimed to capture the attitudes of women in Georgia toward abortion across different gestational ages and to identify the main demographic patterns of those attitudes. We observed that among women in the PASS study, abortion views were variable across gestational age and demographic characteristics. While most women in the sample supported abortion generally (84%), that support decreased as the gestational age increased (31% at 24 weeks). Notably, however, 76% supported abortion at 6 weeks, which is roughly the point at which abortion is restricted in Georgia.

Amongst all the characteristics that we assessed, political orientation, religiosity, and county of residence emerged as the most highly associated with abortion views, which aligns with the findings of prior research [1–5]. Those who identify as moderate or conservative, identify as a religious person, attend religious services more regularly, or live outside of the Atlanta metro area have higher odds of believing abortion should be illegal in every scenario. Religion, politics, and geographic locale are all aspects that, in part, form a person’s identity, which has been found to be particularly influential for abortion views [4,11].

In this study, we observed that although women who were politically liberal, less religious, or lived in Atlanta were at increased odds of believing abortion should be legal in most or all cases, there was substantially variability when asked about specific policies regarding the gestational age at which an abortion occurs. Generally, as the weeks of pregnancy increased, group opinions became less polarized, and the associations between characteristics and abortion views became weaker. Analysis of responses to gestational age questions shows that there is more consensus across religious, political, and geographic groups as pregnancy progresses, specifically at the 24-week mark, which typically aligns with fetal viability. This finding is in line with other research that found support for legal abortion tends to be high, though there is little support for unrestricted abortion access [12].

Furthermore, we found that this sample of women in Georgia tended to overwhelmingly oppose the state’s current policy of restricting abortion beyond six weeks of pregnancy with 76% in favor of abortion being legal at this point. However, this sample is unlikely to be representative of the state as a whole since Atlanta metro residents make up 61% of the sample, though residents of the Atlanta metro area comprise almost half of Georgia’s total population [9]. We also found that those who lived outside of the Atlanta metro area had higher odds of having the view that abortion should be illegal compared to those living inside the Atlanta metro area. Although the Atlanta metro region might be slightly overrepresented in this study and bias our estimates in favor of abortion legality, the city accounts for a significant proportion of the state.

There are limitations to this study. This is a convenience sample of only women recruited using targeted social media ads, which could have led to selection bias with regards to who elected to participate in the study compared to those who did not. An additional limitation to this study has a relatively small sample size. The small sample size limited our ability to make some comparisons and affected our power, resulting in wide confidence intervals. The convenience sampling also resulted in a heavily liberal and Atlanta-based study population, which challenges the generalizability of the results. This also limited our ability to assess factors such as rurality, as even some participants that resided outside of the metro-Atlanta area resided in urban counties. Furthermore, our questions about religion did not capture specific faith or denominational affiliations, so there could be differences across religious groups that are not captured here. Relatedly, our measures of abortion views were obtained from the Pew Research Center which conducts polls, and in that regard these questions are not empirically derived measures of abortion attitudes [13].

A strength of this study is that the timing of the survey captures a period when politics and abortion were common topics of public discourse and news coverage. Furthermore, our study considered a variety of week-based scenarios, which allowed for exploration into the complexity of abortion views across groups. Though not including men in our survey could be considered a limitation, polls have shown that abortion views do not tend to be strongly associated with gender [2]. Thus, it is possible these results might not differ greatly if men were to be included.

## Conclusions

This study found that among a sample of reproductive-aged women in Georgia, there is a majority that is in favor of abortion being legal in most or all cases. This view aligns with preferences for legalized abortion nationally. Attitudes differ mostly along political, religious, and geographic lines. When presented with scenario-based questions, participants across all categories demonstrated more nuanced views around abortion policy. Specifically, abortion at 24 weeks of pregnancy (typically the development of fetal viability) is viewed less favorably by almost every group. In Georgia, however, these views are not reflected by the state-level abortion policy, which is more restrictive than the preferences indicated among most of our sample. Ultimately, policy should reflect scientific evidence, including the need for abortion later in pregnancy.

## Supporting information

S1 Table Associations between demographic, political, religious, and geographic characteristics and abortion views for week-based scenarios, estimated using adjusted logistic regression models. The models are estimating the odds of believing that abortion should be illegal or that “it depends” in this case (vs. abortion should be legal in this case) and are adjusted for age, education status, marital status, and prior pregnancy.
(DOCX)

S2 Table Associations between political, religious, and geographic characteristics and abortion views for week-based scenarios, estimated using unadjusted logistic regression models. The models are estimating the odds of believing that abortion should be illegal or that “it depends” in this case (vs. abortion should be legal in this case).
(DOCX)

S3 Table Distribution of independent variables for week-based abortion view scenarios. (DOCX)
