# Supplementary material for: Determinants of abortion views among reproductive age women in Georgia 2023–2024
Source: PLoS One. 2025 Nov 12;20(11):e0335370. doi: 10.1371/journal.pone.0335370 (PMC12611105; doi:10.1371/journal.pone.0335370)
Supplement: S1 Table — The models are estimating the odds of believing that abortion should be illegal or that “it depends” in this case (vs. abortion should be legal in this case) and are adjusted for age, education status, marital status, and prior pregnancy. (DOCX) [file pone.0335370.s001.docx]

S1 Table: Associations between demographic, political, religious, and geographic characteristics and abortion views for week-based scenarios, estimated using adjusted logistic regression models. The models are estimating the odds of believing that abortion should be illegal or that “it depends” in this case (vs. abortion should be legal in this case) and are adjusted for age, education status, marital status, and prior pregnancy.

|  | Overall | | 6 Weeks | | 14 Weeks | | 24 Weeks | |
| --- | --- | --- | --- | --- | --- | --- | --- | --- |
|  | **OR** | **95% CI** | **OR** | **95% CI** | **OR** | **95% CI** | **OR** | **95% CI** |
| Age | 0.95 | 0.87, 1.03) | 0.99 | (0.92, 1.06) | 0.94 | (0.88, 1.00) | 0.99 | (0.92, 1.06) |
| Race |  |  |  |  |  |  |  |  |
| White | — | — | — | — | — | — | — | — |
| Black | 0.99 | 0.37, 2.65 | 1.06 | 0.44, 2.55 | 3.45 | 1.52, 8.07 | 1.44 | 0.60, 3.50 |
| Non-White | 0.20 | 0.01, 1.15 | 1.46 | 0.48, 4.22 | 3.75 | 1.38, 10.5 | 1.27 | 0.48, 3.53 |
| Ethnicity |  |  |  |  |  |  |  |  |
| Not Hispanic or Latina | — | — | — | — | — | — | — | — |
| Hispanic or Latina | 1.04 | 0.14, 4.99 | 1.07 | 0.21, 4.13 | 0.75 | 0.17, 2.81 | 1.76 | 0.46, 8.62 |
| Marital Status |  |  |  |  |  |  |  |  |
| Married or living with partner | — | — | — | — | — | — | — | — |
| Single | 0.62 | 0.20, 1.82 | 0.70 | 0.27, 1.72 | 0.91 | 0.40, 2.07 | 1.32 | 0.58, 3.05 |
| Household Income |  |  |  |  |  |  |  |  |
| < $54,999 | — | — | — | — | — | — | — | — |
| ≥ $55,000 | 1.41 | 0.46, 4.48 | 1.78 | 0.69, 4.88 | 1.38 | 0.61, 3.15 | 2.07 | 0.92, 4.76 |
| Ever Been Pregnant |  |  |  |  |  |  |  |  |
| Been pregnant before | — | — | — | — | — | — | — | — |
| Never been pregnant | 0.18 | 0.05, 0.54 | 0.29 | 0.11, 0.72 | 0.12 | 0.04, 0.27 | 0.17 | 0.06, 0.42 |
| Employment Status |  |  |  |  |  |  |  |  |
| Unemployed | — | — | — | — | — | — | — | — |
| Employed part or full time | 0.68 | 0.24, 1.95 | 0.68 | 0.29, 1.66 | 0.64 | 0.28, 1.43 | 0.56 | 0.22, 1.32 |
| Student |  |  |  |  |  |  |  |  |
| Not a student | — | — | — | — | — | — | — | — |
| Current student | 0.51 | 0.12, 1.83 | 1.32 | 0.48, 3.65 | 0.65 | 0.26, 1.59 | 0.64 | 0.28, 1.50 |
| Education |  |  |  |  |  |  |  |  |
| No college degree | — | — | — | — | — | — | — | — |
| College degree | 0.66 | 0.24, 1.86 | 0.72 | 0.30, 1.79 | 1.25 | 0.55, 2.86 | 0.97 | 0.39, 2.35 |
| Political Orientation |  |  |  |  |  |  |  |  |
| Liberal | — | — | — | — | — | — | — | — |
| Moderate or Conservative | 10.5 | 3.85, 31.8 | 9.38 | 3.91, 23.9 | 5.66 | 2.38, 14.3 | 4.18 | 1.55, 13.5 |
| Religious Identity |  |  |  |  |  |  |  |  |
| Non-religious person | — | — | — | — | — | — | — | — |
| Religious Person | 2.18 | 0.87, 5.71 | 2.96 | 1.35, 6.73 | 4.00 | 1.93, 8.55 | 1.07 | 0.50, 2.28 |
| Religious Service Attendance |  |  |  |  |  |  |  |  |
| Monthly or less often | — | — | — | — | — | — | — | — |
| Once a week or more often | 5.55 | 2.15, 15.1 | 5.26 | 2.29, 12.5 | 3.80 | 1.72, 8.69 | 2.31 | 0.92, 6.36 |
| County of Residence |  |  |  |  |  |  |  |  |
| Metro Atlanta County | — | — | — | — | — | — | — | — |
| Not in Metro Atlanta | 4.36 | 1.69, 12.4 | 4.07 | 1.83, 9.43 | 2.72 | 1.32, 5.71 | 1.17 | 0.53, 2.59 |
| OR = Odds Ratio, CI = Confidence Interval | | | | | | | | |
