# Supplementary material for: Determinants of abortion views among reproductive age women in Georgia 2023–2024
Source: PLoS One. 2025 Nov 12;20(11):e0335370. doi: 10.1371/journal.pone.0335370 (PMC12611105; doi:10.1371/journal.pone.0335370)
Supplement: S3 Table — (DOCX) [file pone.0335370.s003.docx]

S3 Table. Distribution of independent variables for week-based abortion view scenarios.

|  | **Overall** | | **6 Weeks** | | **14 Weeks** | | **24 Weeks** | |
| --- | --- | --- | --- | --- | --- | --- | --- | --- |
|  | **Legal in most/all cases**  N = 147 | **Illegal in most/all cases**  N = 27 | **Legal in this case**  N = 132 | **Illegal or it depends**  N = 41 | **Legal in this case**  N = 103 | **Illegal or it depends**  N = 70 | **Legal in this case**  N = 53 | **Illegal or it depends**  N = 117 |
|  | **N** | **N** | **N** | **N** | **N** | **N** | **N** | **N** |
| Age |  |  |  |  |  |  |  |  |
| Mean (SD) | 29 (6) | 29 (6) | 28 (6) | 30 (6) | 28 (6) | 30 (6) | 28 (6) | 29 (6) |
| Missing | 1 | 0 | 1 | 0 | 1 | 0 | 1 | 0 |
| Race |  |  |  |  |  |  |  |  |
| White | 67 | 12 | 63 | 17 | 60 | 20 | 28 | 50 |
| Black | 49 | 14 | 44 | 17 | 25 | 37 | 13 | 49 |
| Other | 30 | 1 | 24 | 7 | 17 | 13 | 11 | 18 |
| Missing | 1 | 0 | 1 | 0 | 1 | 0 | 1 | 0 |
| Ethnicity |  |  |  |  |  |  |  |  |
| Not Hispanic or Latina | 133 | 25 | 119 | 38 | 91 | 66 | 48 | 107 |
| Hispanic or Latina | 12 | 2 | 11 | 3 | 10 | 4 | 4 | 9 |
| Missing | 2 | 0 | 2 | 0 | 2 | 0 | 1 | 1 |
| Marital Status |  |  |  |  |  |  |  |  |
| Married or living with partner | 69 | 18 | 60 | 26 | 43 | 43 | 23 | 64 |
| Single | 74 | 9 | 68 | 15 | 57 | 27 | 27 | 53 |
| Missing | 4 | 0 | 4 | 0 | 3 | 0 | 3 | 0 |
| Household Income |  |  |  |  |  |  |  |  |
| < $54,999 | 55 | 9 | 50 | 12 | 41 | 22 | 24 | 37 |
| ≥ $55,000 | 83 | 18 | 74 | 28 | 55 | 46 | 26 | 74 |
| Missing | 9 | 0 | 8 | 1 | 7 | 2 | 3 | 6 |
| Ever Been Pregnant |  |  |  |  |  |  |  |  |
| Been pregnant before | 60 | 21 | 50 | 29 | 30 | 50 | 11 | 68 |
| Never been pregnant | 85 | 6 | 80 | 12 | 71 | 20 | 40 | 49 |
| Missing | 2 | 0 | 2 | 0 | 2 | 0 | 2 | 0 |
| Employment Status |  |  |  |  |  |  |  |  |
| Unemployed | 37 | 8 | 32 | 12 | 25 | 19 | 10 | 34 |
| Employed part or full time | 108 | 19 | 98 | 29 | 76 | 51 | 41 | 83 |
| Missing | 2 | 0 | 2 | 0 | 2 | 0 | 2 | 0 |
| Student |  |  |  |  |  |  |  |  |
| Not a student | 96 | 23 | 87 | 31 | 63 | 56 | 29 | 88 |
| Current student | 50 | 4 | 44 | 10 | 39 | 14 | 23 | 29 |
| Missing | 1 | 0 | 1 | 0 | 1 | 0 | 1 | 0 |
| Education |  |  |  |  |  |  |  |  |
| No college degree | 43 | 10 | 38 | 14 | 30 | 21 | 13 | 37 |
| College degree | 103 | 17 | 93 | 27 | 72 | 49 | 39 | 80 |
| Missing | 1 | 0 | 1 | 0 | 1 | 0 | 1 | 0 |
| Political Orientation |  |  |  |  |  |  |  |  |
| Liberal | 113 | 8 | 106 | 15 | 87 | 34 | 47 | 71 |
| Moderate or Conservative | 25 | 18 | 19 | 24 | 14 | 28 | 5 | 37 |
| Missing | 9 | 1 | 7 | 2 | 2 | 8 | 1 | 9 |
| Religious Identity |  |  |  |  |  |  |  |  |
| Non-religious person | 83 | 9 | 78 | 13 | 69 | 21 | 33 | 56 |
| Religious Person | 57 | 18 | 47 | 28 | 30 | 46 | 19 | 55 |
| Missing | 7 | 0 | 7 | 0 | 4 | 3 | 1 | 6 |
| Religious Service Attendance |  |  |  |  |  |  |  |  |
| Monthly or less often | 118 | 10 | 108 | 18 | 89 | 37 | 46 | 77 |
| Once a week or more often | 28 | 17 | 23 | 23 | 14 | 32 | 7 | 39 |
| Missing | 1 | 0 | 1 | 0 | 0 | 1 | 0 | 1 |
| County of Residence |  |  |  |  |  |  |  |  |
| Metro Atlanta County | 99 | 7 | 92 | 13 | 75 | 30 | 37 | 67 |
| Not in Metro Atlanta | 47 | 20 | 39 | 28 | 27 | 40 | 15 | 50 |
| Missing | 1 | 0 | 1 | 0 | 1 | 0 | 1 | 0 |
